# A Large Kernel Convolutional Neural Network with a Noise Transfer Mechanism for Real-Time Semantic Segmentation

**DOI:** 10.3390/s25175357

**Published:** 2025-08-29

**Authors:** Jinhang Liu, Yuhe Du, Jing Wang, Xing Tang

**Affiliations:** 1 School of Computer Science, Hubei University of Technology, Wuhan 430070, China; liujinhang@hbut.edu.cn (J.L.); 102301202@hbut.edu.cn (Y.D.); 2 Key Laboratory of Green Intelligent Computing Network in Hubei Province, Wuhan 430068, China; 3 School of Computer Science and Artificial Intelligence, Wuhan University of Technology, Wuhan 430070, China; tangxing@whut.edu.cn

**Keywords:** real-time semantic segmentation, large kernel convolution, noise transfer mechanism, position awareness, computer vision

## Abstract

In semantic segmentation tasks, large kernels and Atrous convolution have been utilized to increase the receptive field, enabling models to achieve competitive performance with fewer parameters. However, due to the fixed size of kernel functions, networks incorporating large convolutional kernels are limited in adaptively capturing multi-scale features and fail to effectively leverage global contextual information. To address this issue, we combine Atrous convolution with large kernel convolution, using different dilation rates to compensate for the single-scale receptive field limitation of large kernels. Simultaneously, we employ a dynamic selection mechanism to adaptively highlight the most important spatial features based on global information. Additionally, to enhance the model’s ability to fit the true label distribution, we propose a Multi-Scale Contextual Noise Transfer Matrix (NTM), which uses high-order consistency information from neighborhood representations to estimate NTM and correct supervision signals, thereby improving the model’s generalization capability. Extensive experiments conducted on Cityscapes, ADE20K, and COCO-Stuff-10K demonstrate that this approach achieves a new state-of-the-art balance between speed and accuracy. Specifically, LKNTNet achieves 80.05% mIoU on Cityscapes with an inference speed of 80.7 FPS and 42.7% mIoU on ADE20K with an inference speed of 143.6 FPS.

## 1. Introduction

Semantic segmentation is one of the core tasks in the field of computer vision, aiming to classify each pixel in an image [1], thereby enabling the automatic recognition of objects and understanding their spatial distribution within the image. This technology holds significant application value in areas such as autonomous driving [2,3,4], robotic navigation [5,6], and intelligent transportation systems [7,8,9].

In traditional semantic segmentation methods, Convolutional Neural Networks (CNNs) [10] utilize locally receptive convolutional kernels and pooling operations to effectively extract spatial features from images, enabling pixel level predictions through convolutional layers. CNN-based methods have achieved remarkable progress in improving segmentation accuracy. However, due to their high computational requirements, these methods face challenges in real-time applications, particularly on embedded devices. With the introduction of the Transformer [11] architecture, computational models for semantic segmentation have undergone a revolutionary transformation. Transformers leverage global self-attention mechanisms, overcoming the limitations of traditional CNNs, which focus only on local regions. This allows Transformers to better capture long-range dependencies, thereby enhancing segmentation performance. However, due to the computational complexity of their self-attention mechanisms, Transformers are often more time-consuming than CNNs when processing high-resolution images, resulting in a computational bottleneck.

In recent years, researchers have proposed various lightweight network architectures [12,13,14,15]. But while optimizing accuracy and inference speed, we also observe that street scene images in semantic segmentation applications, such as those in Cityscapes [16], contain a large amount of multi-scale data [17], including cars, roads, and traffic lights. Existing state-of-the-art methods often struggle with blurry or incomplete recognition in fine regions such as road edges and traffic lights. These issues primarily stem from the following factors:

(1) Insufficient capture of receptive fields in current methods, limiting their ability to gather extensive contextual information across multiple scales, which negatively impacts performance in complex scenes.

(2) Inadequate noise-handling mechanisms in existing methods, which can lead to overfitting of neural networks and hinder the generalization of adaptive models.

To address the issue of insufficient receptive fields, RepLKNet [18] proposed a novel method utilizing large kernels, enabling the model to achieve a larger effective receptive field. This improves the preservation of details and segmentation performance in complex scenes, effectively handling challenging elements such as trucks and traffic signs in street scenes, thereby reducing category confusion. Similarly, DSNet [19] extends the large-kernel design concept to Atrous convolution, allowing the network to expand its receptive field and capture richer global information without increasing computational complexity, making it a lightweight alternative to large kernels. By effectively capturing information across different scales [20,21], these approaches help improve the recognition and segmentation accuracy of models in multi-scale scenarios, ensuring better predictions for specific objects at their corresponding scales.

In terms of noise, PSPNet [22] improves the segmentation accuracy of objects in complex scenes by capturing contextual information at different scales, but additional noise suppression strategies may be required in noisy scenes. Specifically, existing methods rely on manually setting thresholds to remove pseudo-labeled samples with low confidence [23,24]; however, due to factors such as differences between different domains, the type of image noise, and the distribution of pixels in each category, the manual threshold setting is usually unable to adapt to all scenarios and it is difficult to ensure that the efficient performance can still be maintained in the presence of severe noise interference.

**Figure 1:**
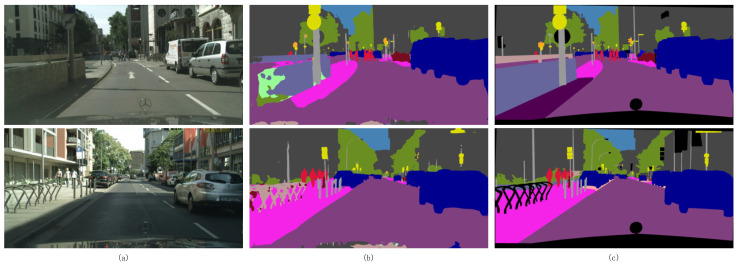
(**a**) Unlabeled target domain data. (**b**) Generated pseudo-labels. (**c**) Ground-truth labels.

Figure 1a illustrates the complexity of an urban street scene, in which there is a wide variety of elements and segmentation is difficult, especially the accurate segmentation of some detail parts is particularly difficult. The practice of simply removing potentially noisy labels by thresholding may lead to misclassification of the target domain, especially in high-confidence cases. Confusing pseudo-labels are usually found in ambiguous categories or poorly represented sub-categories. For example, as shown in Figure 1b, the “low wall” and “road surface”, which have little color difference, are prone to confusion. Additionally, fine objects such as “parking racks” and “street lamps” often exhibit blurred boundaries. These phenomena contribute to the generation of label noise. Simply removing these obfuscated pixels with high noise potential may affect the final segmentation of the model. Therefore, label noise needs to be handled more carefully in order to avoid deleting valid information by mistake and to improve the accuracy of semantic segmentation in the target domain.

To this end, we designed a core module in LKNTNet—Location-Aware Large Kernel (LALK) module to better expand the receptive field and capture multi-scale contextual information. First, the LALK module adopts a multi-level cascade structure of large convolutional kernels (with dilation rates of 1 and 3), effectively integrating spatial information at different scales. Meanwhile, a sigmoid gating mechanism is employed to adaptively regulate the feature responses of each branch. This dynamic control enhances flexibility and mitigates the over-smoothing effect caused by uniform context aggregation, thereby enabling more selective receptive field modeling. In addition, LALK incorporates the Multi-Scale Position-Aware Block (MSPA), which encodes semantic features across various receptive fields through atrous convolution branches. This design enhances the model’s ability to represent small objects and boundary details in complex scenes, significantly improving its sensitivity to edge regions. As a result, the model exhibits more robust performance in scenarios with occlusions, obstructions, and ambiguous boundaries, achieving finer-grained structure recognition. To further improve semantic consistency among features, MSPA innovatively introduces a Modality-Class Token mechanism, constructing a semantic alignment bridge across different modalities. This mechanism aligns features with semantic categories, guiding the network to focus on regions with higher semantic consistency. It effectively reduces classification bias caused by pseudo labels, ambiguous categories, or multi-modal fusion, thereby enhancing semantic discrimination. By fusing the class-level context information with the current features, it guides the aggregation of features to semantic categories, forming a closed-loop optimization link from the underlying features to the high-level semantics. Finally, LALK adopts a fusion design of multi-group convolution and channel attention mechanism. Through a parameter decoupling strategy based on channel grouping, it exponentially increases feature expression diversity while maintaining reduced FLOPs. This approach breaks down inter-group information barriers, building a dual advantage of high computational efficiency and strong representation capability without compromising real-time inference speed, thereby further enhancing the overall performance and generalization ability of the model.

In addition, this paper proposes an innovative structural module—Multi-Scale Contextual Noise Transfer (MSCNT) module. Unlike traditional context encoding or refinement blocks, MSCNT introduces a novel context-driven noise suppression and posterior correction paradigm, specifically designed to address the challenges posed by noisy labels and complex backgrounds. First, MSCNT leverages multi-scale pooling and channel dimensionality reduction to construct a consistent contextual representation. This enables the capture of both global and local semantic information across varying receptive fields, enhancing the model’s structural awareness of target regions. As a result, it effectively separates foreground from background, reduces misclassification rates, and significantly improves reasoning stability, especially in fine-grained target recognition and heavily occluded scenarios. Secondly, the module pioneeringly introduces Noise Transition Matrices (NTM) as a structural output correction mechanism, enabling explicit reconstruction and regulation of the posterior distribution in classification. Unlike fixed-structure attention mechanisms, this approach dynamically adjusts segmentation predictions based on the internal consistency of contextual information. This empowers the model with robust discriminative capability and stability in the face of complex structures, occlusions, background interference, and label noise. Therefore, MSCNT is not merely a feature processing module—it represents a paradigm breakthrough that integrates context modeling, noise recognition, and adaptive correction into a unified framework. It achieves end-to-end systemic innovation, spanning from feature representation to the final decision-making process at the output layer.

**Figure 2:**
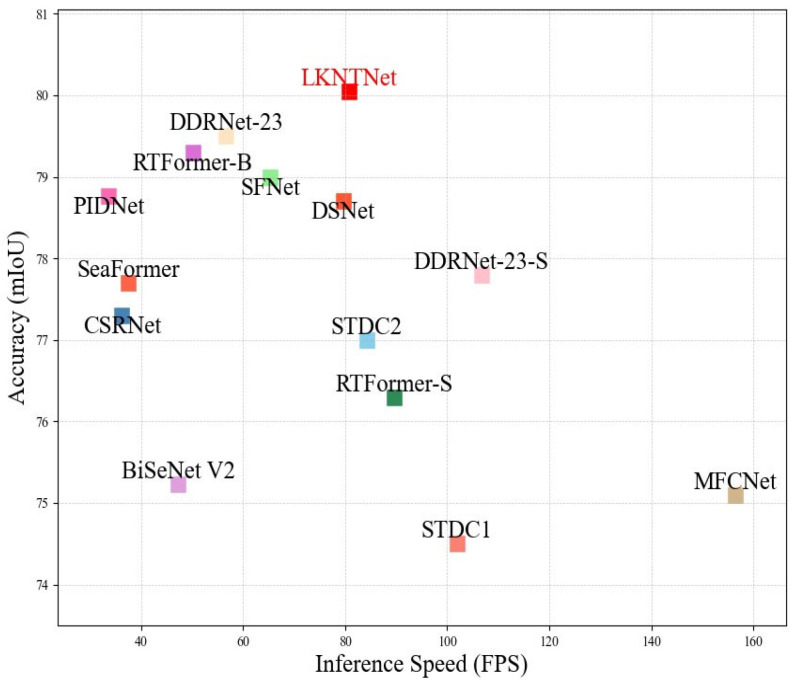
Speed and accuracy performance of different methods on the Cityscapes validation set.

Building upon the two aforementioned modules, we further construct a lightweight semantic segmentation network—LKNTNet. This network achieves unprecedented receptive field expansion and fine-grained semantic discrimination, all while maintaining high inference efficiency. It can efficiently extract semantic information without incurring the heavy computational burden associated with bilateral networks. Specifically, we use a CNN architecture based on Transformer semantic information. This method can effectively extract semantic information and avoid the problem of high computational complexity caused by bilateral networks. LKNTNet learns remote context from training only the Transformer semantic branch to the CNN branch. To reduce the semantic gap between Transformer and CNN, a shared decoder head is used before alignment. During training, the CNN can jointly encode semantic information and spatial details using the aligned semantic information. Thus, LKNTNet can align semantic representations from the large effective receptive field of the Transformer structure while maintaining the efficient inference of the lightweight CNN structure. Figure 2 visualizes the comparison between LKNTNet and other real-time segmentation methods on Cityscapes val. Specifically, the figure is based on the mIoU results of each comparison model on Cityscapes val, where the horizontal axis represents the inference speed (FPS) of the model, and the vertical axis represents the segmentation accuracy (mIoU). Different colors represent different categories of architectures, intuitively showing the trade-off between accuracy and latency for each method. Our method is shown in red, while other methods are represented in blue. The main contributions of this paper are summarized as follows:

(1) Location-Aware Large Kernel (LALK) module: We propose the LALK module, which combines multi-level large kernel convolutions with atrous convolutions, and employs a gating mechanism to achieve dynamic fusion of multi-scale features. This effectively expands the receptive field while suppressing excessive context smoothing. Additionally, a Modality-Class Token mechanism is introduced to facilitate semantic alignment, enhancing the model’s ability to distinguish small objects, boundaries, and ambiguous categories, thereby improving segmentation accuracy and semantic consistency.

(2) Multi-Scale Contextual Noise Transfer (MSCNT) module: The MSCNT module is designed to integrate multi-scale pooling and channel reduction to construct a consistent contextual representation, enhancing the model’s ability to distinguish between foreground and background. Meanwhile, a Noise Transition Matrix (NTM) is introduced to structurally reconstruct and dynamically refine the posterior distribution, effectively improving the model’s discriminative capability and output stability in the presence of noisy labels, occlusions, and complex backgrounds.

(3) Performance Evaluation: We conducted comprehensive evaluations of LKNTNet on multiple datasets, including Cityscapes, ADE20K [25], and COCO-Stuff-10K [26]. The experimental results demonstrate that LKNTNet outperforms existing real-time semantic segmentation methods, achieving a superior balance between accuracy and speed while maintaining real-time performance.

The remainder of this paper is organized as follows: Section 2 provides an overview of related work on the topic. Section 3 presents a detailed description of the LKNTNet architecture, focusing on the design principles of the large-kernel module and multi-scale Atrous convolutions. Section 4 outlines the experimental design and result analysis conducted on datasets such as Cityscapes, ADE20K, and COCO-Stuff-10K, demonstrating the advantages of LKNTNet in real-time semantic segmentation tasks. Finally, Section 5 concludes with a summary of the findings and contributions of this work.

## 2. Materials and Methods

In this section, we discuss four types of methods most relevant to this work: traditional semantic segmentation methods, real-time semantic segmentation methods, Atrous convolutions and large-kernel convolutions, and noisy label processing with noise transition matrices.

### 2.1. Traditional Semantic Segmentation Methods

Semantic segmentation techniques have made significant progress since the advent of convolutional neural network (CNN) models. Initially, research based on Fully Convolutional Networks (FCN) [10] paved the way for pixel level segmentation of images. While FCN employed an end-to-end training approach for pixel-level predictions, its simplistic up-sampling design resulted in blurry edges and insufficient segmentation accuracy. To address these issues, UNet [27] introduced an encoder–decoder structure combined with jump-connected stepwise up-sampling, which significantly improves the segmentation accuracy, and is especially widely used in medical image segmentation. Enhanced models like SegNet [28] further optimized this approach by preserving max-pooling indices for precise up-sampling, reducing redundant features, and improving computational efficiency. The DeepLab series [20] employs Atrous convolutions to expand the receptive field and introduces a fully connected Conditional Random Fields (CRFs) to enhance boundary information, thus improving the segmentation precision. Moreover, PSPNet [22] integrated multi-scale contextual information through a pyramid pooling module, which further enhances the segmentation of complex scenes.

DMANet [29] utilizes multi-scale external attention to process feature maps of different scales and employs a dual-branch cascaded attention mechanism to enable information exchange across branches. FDNet [30] optimizes spectral feature learning by compressing redundant information while highlighting information bands, and can precisely distinguish and enhance high-frequency and low-frequency features. AMKBANet [31] deeply explores and engages with multi-core contextual information containing high-frequency boundary information through the application of multi-core spatial attention and boundary-aware hybrid attention. Unimatch v2 [32] further pushes the performance limits of semi-supervised semantic segmentation through improved matching strategies and consistency constraints, achieving more robust segmentation results in scenarios with limited labeled data. PoCo [33] proposes a pixel-level metric learning paradigm for semantic segmentation by explicitly exploring the structure of labeled pixels.

Although CNN-based models have achieved promising results in many applications, they are generally limited by the locality of convolution operations, making it challenging to effectively capture long-range dependencies and global contextual information. To overcome these limitations, Transformers have been introduced into semantic segmentation in recent years as a complement to CNNs. Vision Transformer (ViT) [11,34] as one of the first works to bring the Transformer architecture into computer vision. By dividing images into fixed-sized patches and leveraging a global self-attention mechanism, ViT effectively captured long-range dependencies. However, its high computational complexity in processing high-resolution images constrained its practical applicability. To address this limitation, SegFormer [35] introduced a lightweight Transformer architecture combined with multi-scale feature extraction methods, which improves the efficiency of semantic segmentation. Nevertheless, SegFormer struggles in complex scenarios, such as fine-grained boundaries or small-object segmentation, primarily due to its insufficient ability to model local details, and the fusion strategy of multi-scale features still has limitations in complex texture scenarios.

### 2.2. Lightweight Real-Time Semantic Segmentation Methods

Early research on real-time semantic segmentation primarily focused on the design of lightweight convolutional neural networks (CNNs) to reduce model parameters and computational complexity, thereby accelerating inference speed. Many methods [36,37] have utilized pre-trained lightweight backbones to tackle real-time segmentation tasks. For instance, BiSeNet V2 [38] employs a dual branch network architecture, extracting shallow detail features and deep semantic features separately, which ensures high inference speed while maintaining segmentation precision. STDC1 [39] effectively reduces the number of parameters and improves the real-time performance through structured deep convolution and adaptive channel selection mechanism, especially in low-resolution scenes. However, when dealing with complex backgrounds and small targets, the accuracy performance may be inferior to that of some more delicate networks. DDRNet-23 [5] incorporates residual modules and deformable convolutional modules, allowing for adaptive adjustment of feature map shapes to address variations across different scenes, which improves the robustness of the model. DMPNet [40] extracts contextual information at different levels and scales in the form of a distributed multi-scale pyramid, enhancing the network’s representational capability. LCFNet [41] introduces a compensation branch to design a three-branch structure for optimizing the network. It selectively extracts features from corresponding branches through two lightweight fusion modules, ultimately achieving multi-branch aggregation with relatively low complexity.

RTFormer [42] leverages the Transformer architecture to capture global features, demonstrating particular advantages in segmenting objects within complex backgrounds and at long distances. AFFormer [43] further improves segmentation accuracy by optimizing attention mechanisms, but its high computational overhead limits its suitability for low-latency applications. SeaFormer [44] combines the strengths of CNN and Transformer architectures, which can better handle global context information. Other hybrid architectures, such as CSRNet [45] and PIDNet [46], enhance recognition and segmentation capabilities by optimizing the fusion of contextual information. However, despite these advances, hybrid architectures often introduce extra attention computation and memory access costs. These methods introduce dense fusion modules between the two branches to enhance the semantic information of the extracted features, leading to increased latency in real-time applications. Moreover, the reliance on Transformer branches for global modeling can be constrained under lightweight settings due to channel compression and reduced spatial resolution. Most existing methods also remain confined to shallow integration between CNN and Transformer components, which limits their ability to fully exploit both local and global representations. In summary, all of these bilateral methods have limitations in terms of inference speed and computational cost due to the additional branches and multiple fusion modules.

### 2.3. Receptive Field Expansion Techniques: Atrous Convolution and Large Kernel Convolution

Traditional convolution operations extract local features by sliding fixed size kernels over an image. However, in certain specific tasks, such as global semantic information modeling or capturing long-range dependencies, the fixed receptive field of traditional convolutions can limit the network’s performance. To overcome the fixed receptive field limitation, researchers have begun exploring convolution operations with dynamic adjustment capabilities. For example, Deformable Convolution Networks (DCN) [47] achieve dynamic receptive field adjustment by learning the offsets of convolution kernels, enabling adaptation to the shapes and sizes of different objects. ACNet [48] further combines the advantages of standard convolution and Atrous convolution, which significantly improves the ability to adapt to complex scenarios by aggregating multi-scale information under different expansion rates. By stacking Atrous blocks, ANet [14] effectively expands the receptive field while maximising the retention of spatial information, thus achieving excellent performance while keeping the number of parameters low.

With advancements in hardware capabilities, Large Kernel Convolution (LKC) has gained significant attention for its applications in semantic segmentation and object detection tasks. ConvNeXt [49] introduced convolutional kernels, substantially enhancing the ability to capture global semantic information while simplifying network structures and reducing reliance on complex attention mechanisms. Subsequently, RepLKNet [18] employed convolutional kernels, demonstrating the potential of convolutional networks in capturing long-range dependency information. Taking this further, ConvFormer [50] achieves a high balance of efficiency and accuracy on multi-tasks by combining the local feature modeling capability of convolution with the global semantic modeling capability of Transformer.

These studies collectively demonstrate that both Atrous convolution and appropriately designed large kernel convolution (LKC) can significantly enhance the extraction of contextual information in deep models. However, most existing approaches treat LKC and Atrous convolution as separate techniques, failing to fully exploit their complementary advantages. To address this limitation, we propose a novel method called LALK (Location-Aware Large Kernel Module), which, for the first time, introduces a position-aware Atrous mechanism within large kernel convolution. This allows the network to not only expand the receptive field but also effectively capture multi-scale semantic details. Unlike static large-kernel methods such as ConvNeXt or RepLKNet, LALK integrates convolutions with varying dilation rates, substantially improving the model’s adaptability to spatial variations and object structures in complex scenes.

### 2.4. Noise Label Processing and Noise Transition Matrix

The problem of learning with noisy labels during training can generally be divided into three classic strategies: label correction [51], loss correction [52,53], and sample reweighting [54]. However, these methods are primarily designed for image-level supervised classification tasks and cannot be directly applied to semantic segmentation. Since semantic segmentation requires predictions for each pixel, the impact of noise is more complex, especially in boundary regions and low-confidence areas. Therefore, improving semantic segmentation performance in the target domain requires careful handling of label noise.

Resampling strategies [55] are another common approach for noise handling, often used to adjust data distribution to mitigate the effects of label noise. However, excessive adjustment through resampling may lead to data distribution bias [56], causing the model to overfit to specific patterns during training and ultimately affecting generalization capability. In the target domain data, if low-confidence regions are excessively removed, it may result in an insufficient number of samples for certain important categories, thereby affecting the final segmentation performance of the model.

The Noise Transition Matrix (NTM) can be used to infer clean class posteriors from noisy class posteriors estimated based on noisy data [57]. It is an essential tool for describing the relationship between clean labels and noisy labels. Early works primarily focused on modeling noisy labels using a known or estimated transition matrix. For example, ref. [58] proposed a loss correction method to enhance the robustness of deep neural networks against noisy labels. However, this method relies on static loss correction and cannot adopt more flexible dynamic noise estimation or noise matrix learning approaches, which limits its performance in complex noisy scenarios.

To address the strong dependency of traditional NTM methods on the noise structure, researchers have proposed various optimization strategies. For example, ref. [59] introduced a method based on maximum likelihood estimation that optimizes the noise matrix directly from the data, reducing the reliance on a prior knowledge. To further enhance the quality of NTM estimation, ref. [60] proposed a framework that jointly optimizes data representation and the noise matrix. By refining the noise matrix to correct label noise, this approach enables the model to adaptively learn the noise distribution without relying on external noise matrix estimation.

These studies demonstrate that appropriate noise-handling strategies can significantly improve a network’s robustness to noisy labels, thereby enhancing segmentation accuracy. To this end, we propose the second original contribution—the Multi-Scale Contextual Noise Transfer (MSCNT) module. This module organically integrates contextual semantic encoding with noise modeling, enhancing the model’s robustness in challenging regions such as low-confidence boundaries. Unlike traditional methods based on static Noise Transition Matrices (NTM), MSCNT introduces a context-driven noise modulation mechanism, enabling flexible noise propagation along the spatial dimension. To the best of our knowledge, this is the first attempt to perform structured noise modeling and dynamic correction for semantic segmentation tasks.

## 3. Proposed Method

**Figure 3:**
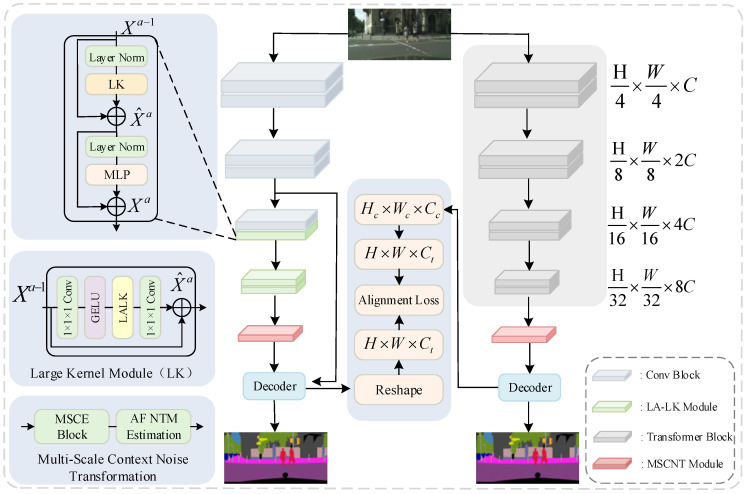
Overview of the LKNTNet architecture.

Removing the semantic branch of dual-branch networks can significantly improve inference speed. However, such simplifications result in shallow single-branch networks lacking sufficient long-range semantic information, leading to a decline in model accuracy. The traditional solution is to restore the accuracy by introducing deeper encoders, more powerful decoders, or complex feature enhancement modules. However, these approaches often substantially increase inference overhead, limiting the speed requirement in practical applications. To tackle this challenge, we propose an improved method to enrich semantic and spatial information without compromising inference speed. Specifically, we introduce LKNTNet, as illustrated in Figure 3. The network is divided into a CNN branch on the left and a training-only Transformer branch on the right, using location-aware big kernel convolution to replace the traditional CNN convolutional layer in the second half of the network, where the LALK Module is the core of the network, which extends the sensory field and captures information at all scales by means of a big convolutional kernel and multiscale context coding. The first two stages are ordinary residual blocks, and the last two stages are location-aware large kernel modules. Then, the features enter the multiscale contextual noise transfer module, where features are extracted using multiscale pooling with different convolutional kernels, and the output in the multiclassification task is adjusted using the transformation matrix, corrected by combining learned posterior distributions, and the inputs are adjusted for convex combinations using the weighting matrix. Finally, the features from network stages 2 and 4 are fed into the decoder, and operations such as Reshape are performed separately from the output of the Transformer decoder, followed by the computation of the alignment loss. The two branches are semantically aligned by the alignment loss in the middle part. In LALK Module, LK Block is the core part.

### 3.1. Location-Aware Large Kernel Module

Traditional CNNs typically rely on small convolutional kernels. While increasing the number of convolutional layers can expand the receptive field (the region of the image that each neuron can “see”) to some extent, this stacking approach has inherent limitations: the receptive field expands slowly, and the computational and memory requirements grow significantly with the network depth. Moreover, convolutional layers at specific levels can only capture features on a single scale, which limits their ability to extract multi-scale features. This is particularly problematic in complex scenes with multi-scale objects, where CNNs often fail to capture small objects and fine details. Meanwhile, the convolutional operations build higher-level representations by aggregating local features, making CNNs adept at capturing local information. However, they often fall short in representing globally consistent features. Consequently, for tasks requiring global contextual information, such as semantic segmentation and scene understanding, traditional CNNs may encounter performance bottlenecks.

**Figure 4:**
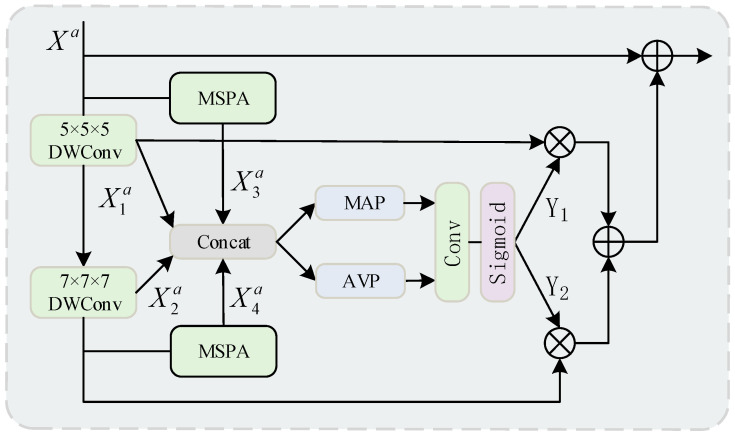
Proposed Location-Aware Large Kernel Module.

The Location-Aware Large Kernel Module (LALK) enhances the expansion of the receptive field by introducing the design of large kernel convolution. This module progressively extends the network’s receptive field through cascaded large kernels, so that the information is gradually aggregated in a larger spatial range to capture richer contextual features. The specific structure of this module is shown in Figure 4.

We introduce two Depthwise Convolution (DWConv) layers into the network, each utilizing kernels with different dilation rates. One convolution layer operates with a dilation rate of 1 and a kernel size of 5 × 5 × 5, while the other uses a dilation rate of 3 and a kernel size of 7 × 7 × 7. The chosen dilation rates allow the convolution kernels to achieve a larger receptive field without increasing computational costs. The outputs of these two large-kernel convolution layers are concatenated with the feature maps from the Multi-Scale Position-Aware (MSPA) block to integrate multi-scale information. This process can be expressed as Equation (1):

(1) $$X_{1}^{a}=DWConv_{\left(5,1\right)}(X^{a})\;X_{2}^{a}=DWConv_{\left(7,3\right)}(X_{1}^{a})\;X_{3}^{a}=MSPA(X^{a})\;X_{4}^{a}=MSPA(X_{2}^{a})$$

Along the channel dimension, we apply Average Pooling (AVP) and Max Pooling (MAP) operations to the concatenated features to capture global information, respectively. Average Pooling is primarily used to extract smooth global features, while Max Pooling focuses on critical high-intensity features. The pooling process can be formulated as Equation (2):

(2) $$W_{avp}=\mathrm{AVP}\left(\left[X_{1}^{a};X_{2}^{a};X_{3}^{a};X_{4}^{a}\right]\right)\;W_{map}=MAP\left(\left[X_{1}^{a};X_{2}^{a};X_{3}^{a};X_{4}^{a}\right]\right)$$

Next, the above concatenated features are fed into a convolutional layer with a kernel size of 7 × 7 × 7 followed by a sigmoid activation function to generate two dynamic selection coefficients Y1 and Y2. These dynamic selection coefficients are used to perform weighted selection on the features output by specific convolutional layers. The selection mechanism can be expressed as Equation (3):

(3) $$\left[Y_{1};Y_{2}\right]=Sigmoid\left(Conv_{7}\left(\left[W_{avp};W_{map}\right]\right)\right)$$

Using the generated dynamic selection coefficients Y1 and Y2, matrix multiplication operations are performed separately on the output of the convolutional layer with kernel size 5 × 5 × 5 and the output of the convolutional layer with kernel size 7 × 7 × 7. This allows for adaptive selection of features for different large kernels and their calibration. Subsequently, the weighted results of the two are summed and further added to the input features, resulting in a more enriched feature representation, the specific process is shown in Equation (4):

(4) $$X^{a}=\left(\left(X_{1}^{a}⊗Y_{1}\right)⊕\left(X_{2}^{a}⊗Y_{2}\right)\right)⊕X^{a}$$

As the kernel size and dilation rate increase layer by layer, the network progressively expands its receptive field while maintaining a low computational cost, thereby capturing richer long-range dependency information. This approach efficiently extends the receptive field, making it particularly suitable for tasks in complex scenarios that require global contextual information.

**Figure 5:**
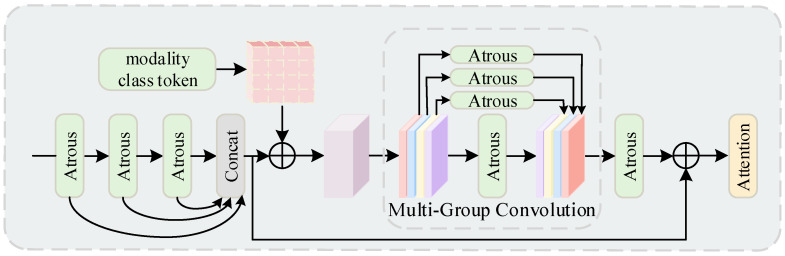
Proposed Multi-Scale Position-Aware Block.

In order to better fuse multi-scale information, we introduced the Multi-Scale Position-Aware Block (MSPA) within the large kernel module, as shown in Figure 5, which processes the input features through multi-layer convolution operations with different dilation rates to extract the feature information at different scales. Specifically, The MSPA block takes the input features and performs three convolution operations with different expansion rates as a precursor module for feature extraction, resulting in three intermediate feature maps. These intermediate feature maps have different receptive fields of 5 × 5, 9 × 9, and 13 × 13, respectively, which effectively capture contextual information at different scales. The intermediate feature maps are concatenated along the channel dimension to form the final multi-scale feature representation. This multi-scale information fusion compensates for the limitation of a single-scale receptive field in large kernels, enhancing the richness of feature representation. The specific process can be formulated as Equation (5).

(5) $$Y_{5\times 5}^{a}=AtrousConv\left(X^{a}\right)\;Y_{9\times 9}^{a}=AtrousConv\left(Y_{5\times 5}^{a}\right)\;Y_{13\times 13}^{a}=AtrousConv\left(Y_{9\times 9}^{a}\right)$$

In semantic segmentation, especially in complex scenes with dense objects or intricate boundaries, features from different categories often overlap or interfere within the same spatial regions. Standard convolution operations may struggle to distinguish these categories, leading to ambiguous feature representations. To address this, we further introduce Modality-Class Tokens (MCT), which serve as the position-aware component. This module is used as a post module to further optimize the feature representation and can provide a unique identifier for each modality class (such as different regions or semantic categories within an image). These identifiers act as a form of inductive bias, helping the model better distinguish and learn the features of different modalities. We initialize the modality class identifier with a truncated cosine function and enable it to inherit gradient properties. Subsequently, the modal class identifiers are dimensionally matched to the feature map via matrix broadcast, thus ensuring that the dimensions of the feature map are aligned with the modal class markers. This combination enhances the feature capture and optimization capabilities of the model while being very adaptable and scalable.

To further enhance the diversity of feature extraction, we introduce Multi-Group Convolutions (MGCs), which divides the feature map into N independent groups. Each group is processed with different convolutional kernels, operating independently without interaction between groups. On this basis, the inter-group information is fused by pointwise convolution, so that the features between different groups can interact effectively. This design allows the network to learn more diverse feature representations, further improving its feature extraction capability. The output feature map of the multi-group convolution is represented as $Y_{i}^{\prime }\in {\mathbb{R}}^{C\times H\times W}$. This structure provides finer characterization for each group of features, enhancing the network’s adaptability to diverse information.

In the final stage of feature fusion, in order to further enhance the effectiveness of the features, we adopt a channel attention mechanism to focus on high-frequency information. Specifically, the calculation process for the channel attention map $A\in {\mathbb{R}}^{C\times 1\times 1}$ is as Equation (6).

(6) $$A=\tau \left(lin\left(act\left(lin\left(\frac{\sum_{i=1}^{H}\sum_{j=1}^{W}Y\left(i,j\right)}{HW}\right)\right)\right)\right)$$

Here, $\tau {(}^{*})$ represents the sigmoid function, $lin{(}^{*})$ represents the linear transformation, and $act{(}^{*})$ represents the activation function. Through the channel attention mechanism, the network can automatically focus on key channel features within the image, amplifying the features that contribute to the task. This enhances the overall representational power of the final features.

In summary, the Location-Aware Large Kernel Module leverages innovative designs such as multi-scale receptive fields, large kernel convolutions, and modality tokens. These enhancements not only expand the receptive field but also improve the expression of multi-scale features and strengthen the network’s ability to model global information effectively.

### 3.2. Multi-Scale Context Noise Transformation

In traditional decoder designs, feature map processing often relies on fixed convolutional kernels or single-scale feature extraction mechanisms, which pose significant limitations in capturing both local and global information. Specifically, when dealing with images containing complex structures and multi-scale features, single-scale feature extraction struggles to balance fine-grained local details and extensive contextual information, leading to suboptimal performance in handling complex visual scenes. Furthermore, when input images contain noise or background interference, traditional decoders lack effective mechanisms to differentiate between useful features and distracting information, which in turn affects the accuracy and robustness of classification. Additionally, traditional decoders typically lack regularization methods, making them prone to overfitting during training. Especially in the case of small datasets or large data diversity, the generalization ability of the model is poor, and it struggles to adapt to new data distributions. These challenges restrict the effectiveness of traditional decoders in real-world applications.

**Figure 6:**
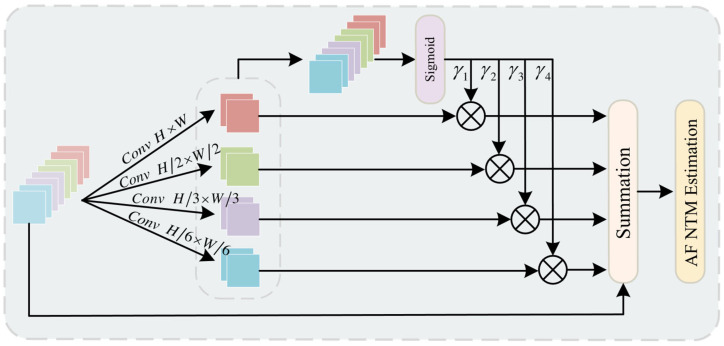
Proposed Multi-Scale Context Noise Transformation Module.

To address the aforementioned limitations, we propose the Multi-Scale Contextual Noise Transfer Module (MSCNT), as shown in Figure 6. The MSCNT module leverages multi-scale pooling and a combination of different convolutional kernels to extract multi-scale features, enabling the capture of information across various spatial scales. This design adapts more effectively to features of different resolutions while preserving rich contextual information. The noise transfer mechanism within the MSCNT module adjusts the outputs of multi-class tasks using a specific transformation matrix, thereby refining the learned posterior distributions to improve classification accuracy. Furthermore, in order to enhance the model’s robustness against noise, we introduce a weighted matrix for convex combination adjustments of input features. This ensures better inter-class differentiation while improving adaptability to complex backgrounds and noisy data, ultimately enhancing the stability of classification predictions.

To extract features more comprehensively, we adopt a multi-branch structure within the MSCNT module, enabling parallel processing of features across multiple scales. Each branch employs convolutional kernels of different sizes to process feature maps, thereby capturing spatial information at various scales. This multi-scale pyramid structure allows each branch to independently learn spatial information at its respective scale and facilitates feature fusion across different resolutions and depths through cross-channel interactions. Specifically, given an input feature map, each branch generates a multi-scale feature map, with the generation function defined as Equation (7).

(7) $$F_{i}=Conv\left(H_{i}\times W_{i}\right)\left(X\right)$$

Here, $H_{i}$ and $W_{i}$ denote the height and width of the i-th convolution kernel, respectively. In this work, the set of convolution kernels used is $\left[H\times W,\frac{H}{2}\times \frac{W}{2},\frac{H}{3}\times \frac{W}{3},\frac{H}{6}\times \frac{W}{6}\right]$, where each kernel has a different size to extract features at multiple scales.

After the convolution operation, the multi-scale feature maps are further upsampled to ensure the consistency of the features at different scales. Subsequently, these contextual feature maps are concatenated to generate a demand coefficient $\gamma =\left[\gamma_{1},\gamma_{2},\gamma_{3},\gamma_{4}\right]\in {\mathbb{R}}^{4\times H\times W}$ that represents the overall contextual information. Demand coefficients are generated using Equation (8).

(8) $$\gamma =\varepsilon \left(W_{2}\mu \left(W_{1}E\right)\right)+1$$

where ε and µ represent the Sigmoid and ReLU functions, respectively, and $E\in {\mathbb{R}}^{\left(4\times C\right)\times H\times W}$ denotes the concatenated upsampled pooling features. $W_{1}\in {\mathbb{R}}^{C\times 4C\times 1\times 1}$ and $W_{2}\in {\mathbb{R}}^{4\times C\times 1\times 1}$ represent 1 × 1 convolution layers. Next, each demand coefficient $\gamma_{i}\in {\mathbb{R}}^{H\times W}$ is multiplied with the contextual features $E_{i}\in {\mathbb{R}}^{C\times H\times W}$ to calibrate the context. Finally, the calibrated context is added element-wise to the original feature map. The specific process is described in Equation (9).

(9) $$M=\sum_{i=1}^{4}\gamma_{i}\cdot P^{n_{i}}\left(X\right)+X$$

where $P^{n_{i}}\left(\cdot \right)$ represents the Spatial Pyramid Pooling layer, and $n_{i}$ denotes the height (or width) of the output size from the pooling layer. By default, n is set to [1,2,3,6].

**Figure 7:**
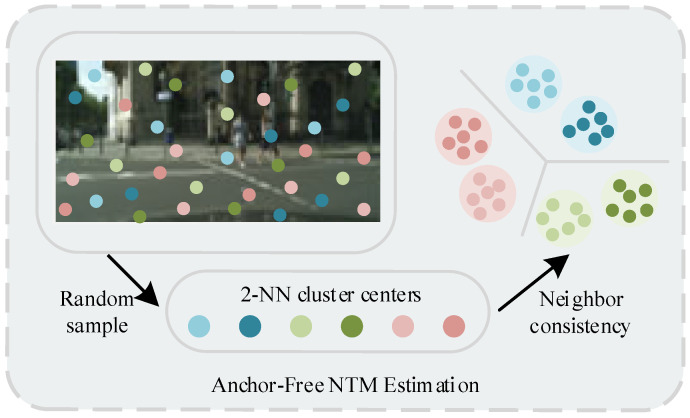
Proposed Anchor-Free NTM Estimation.

In addition, to further enhance the model’s ability to distinguish noisy data, we introduced a k-NN-based noisy label verification mechanism in the MSCNT module, as shown in Figure 7. This mechanism comparing the feature representations of noisy labels with their k-NNs to discriminate pixels with corrupted labels ensures discrimination between categories and also improves the robustness of classification predictions. It looks at the k nearest neighbors of the target feature in the dataset and uses their class information to determine the category of the new feature. Since we lack prior knowledge about whether the labels are clean, we set k to 2 for voting and use the majority class as the ground truth semantics. A similar idea has already been mentioned in [61]. Suppose the input noisy label is $\tilde{k_{1}}$, and its nearest and second-nearest neighbors among the k-NN are labeled as $\tilde{k_{2}}$ and $\tilde{k_{3}}$, respectively. If these two labels are inconsistent, it may indicate that the target pixel’s label is incorrect. Due to k-NN clustering, their ground truth labels are equal, i.e., $\tilde{k_{1}}=\tilde{k_{2}}=\tilde{k_{3}}$. For this purpose, we define $F={\left[f\left(k=i\right)\right]}^{T},i\in \left[1,C\right]$, and then the probability equation as in Equation (10) always holds.

(10) $$f\left(\tilde{k_{1}}=j\right)=\sum_{i=1}^{C}f\left(\tilde{k_{1}}=j\left|k_{1}=i\right.\right)f\left(k_{1}=i\right)=\sum_{i=1}^{C}T_{ij}F_{i}$$

When this condition is satisfied, the target label is considered reliable; otherwise, it is marked as a noisy label. Further, we can utilize the second-order consistency equation as in Equation (11).

(11) $$\begin{array}{l} f\left(\tilde{k_{1}}=j_{1},\tilde{k_{2}}=j_{2}\right)\\ =\sum_{i=1}^{C}f\left(\tilde{k_{1}}=j_{1},\tilde{k_{2}}=j_{2}\left|k_{1}=i,k_{2}=i\right.\right)f\left(k_{1}=k_{2}=i\right)\\ =\sum_{i=1}^{C}f\left(\tilde{k_{1}}=j_{1}\left|k_{1}=i\right.\right)f\left(\tilde{k_{2}}=j_{2}\left|k_{2}=i\right.\right)f\left(k_{1}=i\right)\\ =\sum_{i=1}^{C}T_{ij_{1}}T_{ij_{2}}F_{i} \end{array}$$

Subsequently, the k-NN corresponding to the noisy label $\tilde{y_{s}}$ is obtained. Specifically, the feature representation extracted by the backbone network is used to compute the cosine similarity to confirm the neighbor labels ${\tilde{y}}_{s_{1}}$ and ${\tilde{y}}_{s_{2}}$. Then, the expected mean of a set of sampled instances R from the noisy dataset is used to estimate each higher-order consistency. The specific process is described in Equation (12).

(12) $$\eta^{1}=\frac{1}{\left|S\right|}\sum_{s=1}^{S}\xi \left\{\tilde{k_{s}}=i\right\}\;\eta_{m}^{2}=\frac{1}{\left|S\right|}\sum_{s=1}^{S}\xi \left\{\tilde{k_{s}}=i,\tilde{k_{s_{1}}}=i+m\right\}\;\eta_{m,n}^{3}=\frac{1}{\left|S\right|}\sum_{s=1}^{S}\xi \left\{\tilde{k_{s}}=i,\tilde{k_{s_{1}}}=i+m,\tilde{k_{s_{2}}}=i+n\right\}$$

Here, $\xi {\{}^{*}\}$ denotes the indicator function, which equals 1 if the condition is satisfied and 0 otherwise. S is randomly sampled following a uniform distribution, and performs consistency calculations multiple times, while |S| represents the sample size.

The Noise Transfer Matrix (NTM) corrects model predictions by explicitly modeling the noise distribution of labels in the training data. Specifically, NTM can smooth the model’s predictions on noisy labels, preventing the model from directly fitting incorrect labels, thereby effectively alleviating overfitting issues. In this way, NTM enables the model to consider the impact of label noise during the training phase, thereby learning more robust feature distributions that are closer to the true labels. This not only improves the model’s stability on noisy data but also significantly enhances its generalization performance on clean validation data.

### 3.3. Alignment Loss

To achieve effective semantic alignment between the Transformer branch and the CNN branch, we propose a specialized alignment loss function. This alignment loss focuses on preserving the consistency of semantic information, thereby enhancing the expressive capability of the model. Specifically, we employ a Channel-Wise Distillation Loss (CWDL) [62] to achieve the alignment effect. The core of CWDL lies in unifying the channel dimensions of the features, enabling features from the Transformer branch and the CNN branch to be aligned and compared within the same feature space. This facilitates the sharing and calibration of semantic information across different network structures.

Before calculating the alignment loss, it is necessary to preprocess the features from the Transformer and CNN branches to ensure consistency in spatial resolution and channel dimensions. The specific operations include up-sampling and down-sampling. At the initial stage, the features from the Transformer branch generally have a higher density of semantic information but lower spatial resolution. In contrast, the features from the CNN branch retain more spatial detail but exhibit a lower level of semantic abstraction. Therefore, we first perform an up-sampling operation on the Transformer branch features to increase their spatial resolution, aligning them with the spatial scale of the CNN branch features. Similarly, for the CNN branch features, we apply down-sampling to reduce spatial information, making them better aligned with the high-level semantic features of the Transformer branch.

To preserve the original information of the CNN branch during feature fusion, we designed a feature projection module that projects the features of the CNN branch onto the dimensions of the Transformer branch to achieve channel alignment. Specifically, the representative features from the second and fourth stages of the CNN branch are selected, and these features are processed through pointwise convolution to expand their channel dimensions, ensuring consistency between the feature dimensions of the CNN and Transformer branches. Pointwise convolution not only adjusts the number of feature channels but also aligns the features while maintaining their content, thereby avoiding the loss of detail in the CNN branch features during the dimensional transformation process.

After completing the alignment of spatial and channel dimensions, the features from the CNN branch are semantically aligned with those from the Transformer branch. The high-dimensional features of the CNN branch are fed into the Transformer decoder to further enhance the semantic representation of the features and facilitate feature fusion in the high-dimensional feature space. During this process, the semantic alignment loss is used to measure the feature discrepancy between the Transformer branch and the CNN branch. Finally, semantic alignment loss is applied to the projected features to align their semantic representations. The aligned features are integrated into the Transformer decoder, participating in the decoding process and contributing to the final segmentation prediction. This design ensures that the advantages of alignment are not limited to intermediate representation learning, but also directly improve the quality of the model’s final output.

Specifically, the core design of the semantic alignment loss function focuses on aligning the feature maps generated by the two network architectures. Assuming the number of channels is c=1,2,…,C and the spatial dimensions are W⋅H, the feature maps from the Transformer branch and the CNN branch are denoted as $X_{t}$ and $X_{\delta }$, respectively. The t denotes a hyper-parameter called temperature. And the larger t is, the softer the probability distribution is. Then the alignment loss is calculated as shown in Equation (13):

(13) $$\psi \left(X_{c}\right)=\frac{\exp\left(\frac{X_{c},i}{t}\right)}{\sum_{i=1}^{W\cdot H}\exp\left(\frac{X_{c},i}{t}\right)}\;L_{cwd}=\frac{t^{2}}{C}\sum_{c=1}^{C}\sum_{i=1}^{W\cdot H}\psi \left(X_{t}^{c,i}\right)\cdot \log\left[\frac{\psi \left(X_{t}^{c,i}\right)}{\psi \left(X_{\delta }^{c,i}\right)}\right]$$

To further enhance the effectiveness of the alignment loss, we adopt different feature fusion strategies at various training stages. During the early training phase, the model’s ability to capture local features is still underdeveloped. Therefore, we primarily focus on aligning low-level features, using feedback from the loss function to guide the consistency between the CNN and Transformer branches in local feature representation. In the later training phase, as the semantic information becomes richer and the understanding of global context deepens, we gradually shift attention to aligning high-level semantic features to improve the model’s ability to capture global information. This progressive feature fusion strategy better aligns with the model’s learning process, ensuring that the alignment loss effectively optimizes the model at different stages of training.

### 3.4. Decoder Head

The decoder head is composed of a DAPPM module [5] and a segmentation head. To further enhance contextual information, we introduce an additional DAPPM after the output of MSCNT module. The resulting feature map is then concatenated with the feature map from Stage 2. The fused feature is subsequently fed into the segmentation head, which consists of a 3 × 3 convolution followed by batch normalization and a ReLU activation, and finally a 1 × 1 convolution is applied for pixel-wise classification.

## 4. Experiments and Discussion

In this section, we first introduce the evaluation setup, including the datasets and methods used for comparison. Then, we compare the algorithm with state-of-the-art semantic segmentation methods on challenging benchmark datasets such as Cityscapes, ADE20K, and COCO-Stuff-10K.

### 4.1. Datasets

(1) Cityscapes: This dataset is a high-quality urban street view dataset provided by Germany, consisting of 5000 finely annotated images and 20,000 coarsely annotated images, covering more than 50 European cities. The images have a resolution of 2048 × 1024 pixels and include detailed pixel level annotations categorized into 30 classes, of which 19 are used for semantic segmentation tasks. These classes represent common urban scene elements, such as roads, pedestrians, vehicles, buildings, and trees. For training, we set the initial learning rate to 0.004 and a weight decay of 0.0125, using the AdamW [63] optimizer. Specifically, we employ a polygon learning strategy with a power of 0.9 and implement a data expansion method that includes random cropping, random scaling, and random horizontal flipping. Random cropping of 1024 × 1024 and scaling in the range of 0.5 to 2.0 were used. The model was trained over 160 k iterations with a batch size of 16.

(2) ADE20K: This dataset, provided by MIT, contains over 25,000 images of various scenes and scales, covering a wide range of environments, including indoor and outdoor settings. The image resolutions vary, but the average size is approximately 512 × 512 pixels. ADE20K offers detailed pixel-level annotations spanning 150 categories, including buildings, roads, furniture, plants, animals, and more. We set the initial learning rate to 0.0005, with a random crop size of 512 × 512, and applied scaling in the range of 0.5 to 2.0. The model was trained for 160k iterations with a batch size of 32. All other training details were the same as those used for the Cityscapes dataset.

(3) COCO-Stuff-10K: This dataset is an extended version of the COCO dataset, comprising 10,000 images that cover a wide range of scenes and object categories. Each image includes pixel-level annotations for 172 classes, which are divided into “stuff” categories (e.g., grass, sky, water) and “thing” categories (e.g., people, vehicles, furniture). The dataset provides detailed segmentation masks for each scene, annotating not only objects but also background and environmental elements. We set the initial learning rate to 0.01 and the weight decay to 0.00006, using the AdamW optimizer for training. Random cropping with a size of 640 × 640 was applied, along with random scaling in the range of 0.5 to 2.0. All other training details were the same as those used for the Cityscapes dataset.

### 4.2. Evaluation Metrics

To evaluate the semantic segmentation performance of the data, we primarily use the mean Intersection over Union (mIoU) as the evaluation metric. mIoU is a widely used evaluation metric in semantic segmentation tasks. It quantifies the degree of overla between the predicted segmentation and the ground truth segmentation by computing the ratio of the intersection to the union of the two sets of pixels. The mIoU score is obtained by averaging the IoU scores across all classes in the dataset. It provides a measure of the overall accuracy of the segmentation model. In addition, the inference speed is reported in terms of frames per second (FPS), and the complexity of the model is evaluated based on the number of parameters (Params) and giga floating-point operations (GFLOPs). In this context, mIoU can effectively enable a fair comparison between the proposed LKNTNet and other state-of-the-art methods. The formula for this evaluation indicator is shown in Equation (14).

(14) $$mIoU=\frac{1}{N}\sum_{n}^{N}\frac{P_{t}}{S_{n}}$$

Here, $S_{n}=P_{t}+P_{f}+N_{f}$ and $P_{t}$ denote the number of true positives, $P_{f}$ denotes the number of false positives, and $N_{f}$ denotes the number of false negatives, all of which are specifically related to each foreground category indexed by n.

### 4.3. Implementation Details

All the experiments in this study were conducted using the PyTorch framework (version 1.12.1) and Python 3.8.10. The code was trained and tested on a server equipped with a single NVIDIA GeForce RTX 4090 GPU (16 GB memory) (Nvidia, Santa Clara, CA, USA) and running the Ubuntu 20.04 LTS operating system, using the PyCharm IDE (Professional Edition 2023.1). Additional dependencies include PyTorch 1.11.0, TorchVision 0.12.1, and mmcv-full 1.6.0.

### 4.4. Performance Comparison

In this study, we conducted a comparative analysis of the method against several representative and state-of-the-art semantic segmentation approaches.

(1) Performance Comparison on the Cityscapes Dataset: Comparison of the synthesis and classification of LKNTNet with other methods is shown in Table 1 and Table 2. As shown in Table 1, proposed LKNTNet achieves the best results in terms of mIoU, while also demonstrating the optimal speed accuracy trade-off in TensorRT and Torch implementations. We achieve a state-of-the-art real-time segmentation performance with 80.05% mIoU at 80.7 FPS. Compared to the second-best method, LKNTNet improves mIoU by 0.58%. Recent works, such as BiSeNet V2, utilize a dual-branch structure comprising a spatial branch and a context branch with lightweight convolutional operations. This approach enhances computational efficiency while maintaining segmentation accuracy. DDRNet employs dynamic residual modules that automatically adjust residual connections based on input features, significantly improving global feature extraction, particularly in multi-scale feature fusion and local detail processing. Transformer-based segmentation methods like RTFormer focus on global information extraction, effectively capturing long-range dependencies, making them particularly suited for complex scene segmentation tasks. AFFormer combines convolutional and Transformer, introducing an adaptive attention mechanism to fuse local and global information, which is advantageous in scenarios with ambiguous boundaries. PIDNet adopts staged feature fusion and depthwise separable convolution modules, optimizing the network’s performance in multi-scale feature handling. LCFNet incorporates a local context feature fusion mechanism along with a global context module, balancing global and local information capture to enhance segmentation performance. DSNet leverages dual-scale feature fusion and adaptive convolution modules to improve segmentation precision for fine details, particularly excelling in boundary handling.

**Figure 8:**
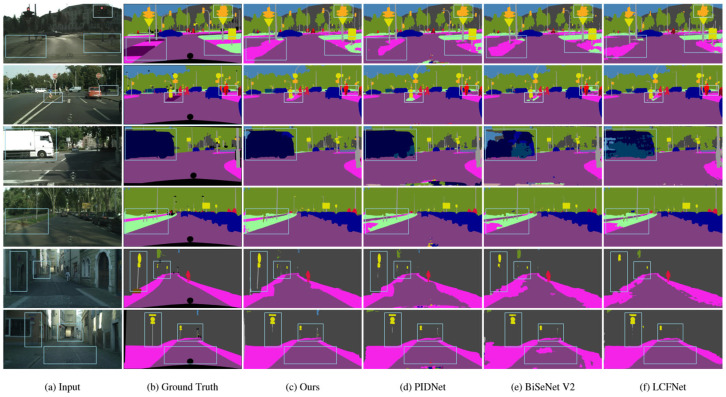
Visualization results on the Cityscapes validation set. Compared to other methods, the generated masks exhibit finer details, as highlighted in the light blue boxes.

To provide an intuitive comparison of the segmentation performance of different algorithms, we present the segmentation results on the Cityscapes dataset in Figure 8. LKNTNet demonstrates outstanding performance in handling texture details and boundaries, especially in areas with complex boundaries such as streetlights, poles, and road surfaces, achieving clearer and more accurate segmentation. This showcases its exceptional separation capability. Specifically, the LALK module in LKNTNet utilizes larger convolution kernels to provide the network with a broader receptive field, while the Atrous convolutions in the MSPA module capture multi-scale contextual information, effectively enhancing the network’s modeling capacity. Moreover, the MSCNT module extracts multi-scale features and employs a noise transition mechanism to distinguish between different categories, producing more complete and well-defined segmentation results. It can be seen that in the labeled region, LKNTNet has a better recognition effect with more complete and neat boundaries.

(2) Performance Comparison on ADE20K Dataset: The experimental results on the ADE20K dataset further validate the outstanding performance of LKNTNet, similar to the results on the Cityscapes dataset. As shown in Table 3, LKNTNet achieves the best accuracy at the fastest speed, reaching an mIoU of 42.7% at 143.6 FPS. Considering the diverse range of images and semantic categories in ADE20K, this remarkable result further demonstrates the generalization capability of LKNTNet.

(3) Performance Comparison on the COCO-Stuff-10K Dataset: As shown in Table 4, LKNTNet maintains the highest inference speed among real-time semantic segmentation methods on the COCO-Stuff-10K dataset. With an input size of 640 × 640, LKNTNet achieves an mIoU of 35.4% at 143.6 FPS.

### 4.5. Ablation Study

#### 4.5.1. Validate the Effectiveness of Different Modules

The proposed LKNTNet comprises three core components: the Location-Aware Large Kernel Convolution (LALK), the Multi-Scale Position-Aware Module (MSPA), and the Multi-Scale Contextual Noise Transfer Module (MSCNT). To validate the effectiveness of each component, we designed a series of ablation experiments by systematically removing or adding specific components for analysis. As shown in Table 5, we conducted four ablation experiments to systematically evaluate the contribution of each module. In the first experiment, the complete LALK module was retained while the MSCNT module was removed. In the second experiment, the LALK module was replaced with a traditional CNN branch while retaining the MSCNT module to assess its impact on segmentation accuracy. The third experiment added the MSCNT module and removed the MSPA module based on the first experiment to further evaluate the contribution of the MSPA module to segmentation performance. As illustrated by the results in Table 5, each core component is critical for enhancing LKNTNet’s performance. Specifically, the LALK module significantly improves the receptive field while capturing multi-scale semantic information. The MSCNT module enhances classification accuracy and reduces overfitting.

To further verify the improvements brought by NTM alone and the differences in improvements after integrating it with the MSCE module, we conducted additional ablation experiments. To ensure consistency in experimental conditions, we retained the complete LALK and MSPA modules as the baseline architecture. In the first experiment, we kept only the MSCE module while removing the NTM module to evaluate the independent performance of the MSCE module. In the second experiment, we retained only the NTM module while removing the MSCE module to assess the impact of the NTM module alone. Through these experiments, we were able to clearly compare the contributions of NTM and MSCE to segmentation accuracy.

**Figure 9:**
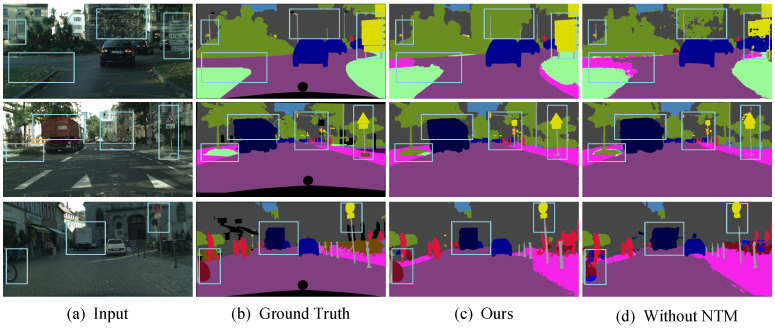
Visualization of NTM module ablation experiments.

From the results in Table 6, it can be seen that removing either the MSCE module or the NTM module alone leads to a decrease in segmentation accuracy, indicating that both play complementary roles within the MSCNT module. Notably, compared to Experiment 4 in Table 5, the synergy between the MSCE and NTM modules significantly improves segmentation performance. At the same time, we observed that although the gradual introduction of various modules into the network inevitably leads to a certain degree of decline in inference speed, this overhead is completely acceptable in comparison to the significant improvement in segmentation accuracy. In other words, while these modules increase the computational load, they significantly enhance the model’s ability to characterize complex scenes and fine-grained targets, thereby achieving a good overall performance in terms of accuracy–efficiency balance. The combination of NTM and MSCE not only enhances the model’s robustness to noise but also improves its overall stability. Specifically, the NTM module plays a crucial role in denoising and adaptive noise adjustment, while the MSCE module further optimizes segmentation results through multi-scale contextual information capture and noise transition. To more intuitively demonstrate the effectiveness of the proposed MSCNT module in suppressing prediction noise, we have added a visual comparison of the segmentation results before and after NTM correction in Figure 9. These visual results clearly reflect the role of this module in reducing prediction noise and optimizing boundary details.

To gain a deeper understanding of the independent contribution of the NTM module, we conducted additional experiments to explore its performance under different noise levels. Specifically, the low-noise setting was defined as adding Gaussian noise with a standard deviation of 0.01, while the high-noise setting used a standard deviation of 0.05. These values were selected to simulate mild and severe noise interference in practical scenarios. We evaluated the NTM module in both environments, and as shown in Table 7, the results demonstrate that the NTM module effectively suppresses noise interference under high-noise conditions, significantly improving segmentation accuracy.

Combining the above experimental results, we can conclude that the NTM module and the MSCE module complement each other and jointly improve the performance of semantic segmentation. The organic combination of the two enables the MSCNT module to show stronger robustness and accuracy in complex noise environments.

To more intuitively demonstrate the advantages of DWConv in this model, we replaced it with the conventional standard convolution (Conv) for comparative experiments. This allows us to more clearly observe the differences between the two in terms of model performance. To ensure the consistency of experimental conditions, we kept all other modules and the underlying architecture unchanged, ensuring the accuracy and comparability of the results.

As shown in Table 8, using DWConv can improve mIoU, making the model more efficient. Additionally, this optimization method preserves the feature representation capability while enhancing the model’s applicability in realtime scenarios, further validating its advantages in semantic segmentation tasks.

#### 4.5.2. Ablation Study on Semantic Alignment Loss

We conducted a systematic experimental analysis on the types, application positions, and weight configurations of semantic alignment losses. The proposed semantic alignment loss is designed to guide CNNs to more effectively learn long-range semantic information from Transformers. Therefore, loss functions that can effectively supervise contextual information learning are more suitable for use as alignment losses.

We selected Kullback–Leibler Divergence Loss (KL Loss) [64], Mutual Information Loss (MI Loss) [65], L2 Norm Loss (L2 Loss) [66], and CWD Loss for these experiments. From the results in Table 9, it can be seen that CWD Loss has a significant positive impact on model performance, whereas L2 Loss performs worse than the Baseline. Meanwhile, KL Loss exhibits almost the same performance as the Baseline before calibration. This may be because the advantage of CWD Loss lies in its adaptive alignment of channel distributions. Compared to L2 and KL Losses, which align feature maps in a per-element manner, CWD Loss transforms feature activations into channel-wise probability distributions, allowing for better alignment of overall channel-level information and capturing richer global semantic context. This approach not only avoids the information loss caused by the rigid alignment (i.e., hard alignment) of L2 Loss but also overcomes the alignment bias that KL Loss may introduce in an uncalibrated state. Additionally, CWD Loss maintains robustness across different scales of feature spaces, enabling long-range information from the Transformer to be more naturally transferred to the CNN branch, thereby enhancing the overall alignment effectiveness of the model.

Furthermore, the MI loss uses the variational estimation technique from the VID method [67] to measure the correlation between the channel distributions, which performs similarly to the CWD loss. The commonality between the two lies in aligning channel distributions rather than local feature points, indicating that in LKNTNet, modeling information at the channel level is more critical than the specific form of similarity measurement. This is because the goal of LKNTNet is to align long-range contextual information in semantic branches, which is mainly reflected in the overall channel distribution rather than in local pixels or regions. Therefore, compared to traditional loss functions, CWD Loss can more effectively leverage global channel information, facilitating more efficient cross-modal feature alignment.

Additionally, Table 10 analyzes the impact of the application position of semantic alignment loss on performance. The experimental results indicate that applying semantic alignment loss to the second and fourth layers of features, the decoder, and the output logits achieves the optimal mIoU performance configuration. Notably, the application position of the alignment loss has no significant impact on inference FPS.

Table 11 presents the experimental results on the weights of alignment loss. For output logits, a weight of 3 was identified as the optimal setting [67]. For feature alignment, a weight of 15 yielded the best performance. Smaller weights fail to fully exploit the high-quality semantic information provided by the Transformer, while larger weights conflict with the CE loss, degrading model performance. Moreover, when the weight is excessively large (e.g., 30), the training loss fluctuates significantly during the process, leading to a substantial drop in model accuracy.

#### 4.5.3. Ablation Study on Convolution Kernel Size

To further analyze the impact of convolution kernel size on performance in the proposed module, we conducted ablation experiments on different convolution kernel configurations. Table 12 shows the experimental results for different convolution kernel sizes. As shown in the table, when the convolution kernel size is set to 5×5×5+7×7×7, the model achieves the highest mIoU on the benchmark dataset, improving by 0.8% compared to the 5×5×5+5×5×5 kernel, but simultaneously introducing approximately 0.5% reduction in inference speed. Compared to the 7×7×7+7×7×7 kernel, it improved by 1%, also increased inference speed by approximately 3%. When the convolution kernel size is further increased, performance improvements tend to plateau, while incurring unnecessary computational costs. Therefore, in all experiments in this paper, the convolution kernel size is fixed at 5×5×5+7×7×7 to achieve the optimal balance between accuracy and efficiency. It is worth noting that, despite being slightly slower in speed, the significant improvement in accuracy demonstrates the effectiveness of large receptive fields in modeling contextual relationships.

### 4.6. Model Complexity Analysis

We evaluate the computational complexity of the proposed LKNTNet using the following metrics: the number of model parameters (Params) and frames per second (FPS). Params assesses the memory requirements, while FPS measures the execution speed of the model. Ideally, an efficient model should exhibit a smaller Params value while maintaining a higher FPS value.

Table 1 demonstrates that LKNTNet achieved a segmentation accuracy of 80.05% mIoU, the best among all methods. This excellent performance is attributed to the fact that LKNTNet introduces a large kernel convolution module for extending the receptive field and uses a noise transfer mechanism to improve the model’s generalization ability, which results in accurate capture of contextual information. These designs provide the model with powerful feature representation and computational efficiency for processing complex scene data. In addition, the proposed LKNTNet exhibits lower Params than PIDNet and STDC2 and better performance than BMSeNet and DSNet in terms of FPS. LKNTNet embodies high computational efficiency and accuracy balancing ability, demonstrating the superiority of realizing high-precision real-time semantic segmentation under the condition of keeping low computational complexity.

## 5. Conclusions

This paper proposes a novel dual-branch network, LKNTNet, which expands the receptive field through multi-scale perception to enhance both global and local information modeling capabilities. Specifically, we integrate large kernel convolutions and Atrous convolutions to effectively expand the receptive field at different scales, thus capturing richer contextual information. Additionally, we estimate the NTM and correct the supervision signals using high-order consistency information from neighbor representations, eliminating the need for precisely defined anchors. Experimental results on multiple benchmark datasets demonstrate that LKNTNet achieves superior performance in both accuracy and computational efficiency. For example, on the Cityscapes dataset, LKNTNet achieves 80.05% mIoU; on the ADE20K dataset, it reaches 42.7% mIoU, outperforming several state-of-the-art methods; and on the COCO-Stuff-10K dataset, it also performs exceptionally well, achieving 35.4% mIoU. These results validate the effectiveness of this approach in complex scenes while highlighting the potential of LKNTNet for efficient semantic segmentation tasks.

## Figures and Tables

**Table 1:** Comparison of accuracy and speed with other state-of-the-art real-time methods on the Cityscapes validation set. “-” indicates that the corresponding results are not provided by the method. Our results are highlighted in red. # Params refers to the number of parameters.

Method	Reference	# Params	Resolution	GLOPs	FPS	mIoU (%)
BiSeNet V2	CVPR2020	-	2048 × 1024	118.5	47.3	75.23
STDC2-Seg75	CVPR2021	22.2 M	1536 × 768	-	84.3	77.0
SegNet-T-Seg100	NeurIPS2022b	4.3 M	2048 × 1024	-	28.1	79.8
DDRNet-23	TIP2022	20.1 M	2048 × 1024	143.1	56.7	79.5
RTFormer-B	NeurIPS2022	16.8 M	2048 × 1024	-	50.2	79.3
AFFormer-B-Seg	AAAI2023	3.0 M	2048 × 1024	-	28.4	78.7
SeaFormer-B-Seg100	ICLR2023	8.6 M	2048 × 1024	-	37.5	77.7
CSRNet-heavy	ESWA2023	-	768 × 768	-	36.3	77.3
PIDNet-M	CVPR2023	42.8 M	2048 × 1024	197.4	33.7	78.76
LCFNet	TransIntellVeh2024	8.3 M	2048 × 1024	77.92	87.6	68.67
DSNet	CVPR2024	7.6 M	2048 × 1024	226.6	79.6	78.7
Ours		20.5 M	2048 × 1024	158.7	80.7	80.05

**Table 2:** Classification results on the Cityscapes validation set (IoU scores). The category list (from left to right): road (Roa), sidewalk (Sid), building (Bui), wall (Wal), fence (Fen), pole (Pol), traffic light (Tli), traffic sign (TSi), vegetation (Veg), terrain (Ter), sky (Sky), person (Per), rider (Rid), car (Car), truck (Tru), bus (Bus), train (Tra), motorcycle (Mot), and bicycle (Bic). The best results in each category are highlighted in red.

Method	Roa	Sid	Bui	Wal	Fen	Pol	TLi	TSi	Veg	Ter
BiSeNet V2	97.95	83.36	91.77	50.16	57.27	62.02	68.62	76.41	92.24	62.18
PIDNet	98.10	84.91	92.90	56.63	63.53	65.48	71.98	78.49	92.61	64.91
LCFNet	97.63	81.71	90.54	53.76	53.82	59.15	56.76	69.72	91.52	59.13
Ours	98.54	87.68	92.83	52.19	65.34	66.83	73.42	80.84	92.49	65.74
Method	Sky	Per	Rid	Car	Tru	Bus	Tra	Mot	Bic	mIoU
BiSeNet V2	94.57	81.12	58.32	94.65	72.07	79.86	75.03	55.25	76.52	75.23
PIDNet	94.66	82.62	65.49	95.30	80.80	88.44	79.75	62.73	77.07	78.76
LCFNet	93.50	76.42	51.25	92.54	49.59	65.37	45.45	45.62	71.23	68.67
Ours	94.84	83.17	65.26	95.67	86.05	89.75	84.22	67.04	79.09	80.05

**Table 3:** Accuracy and Speed Comparison with Other Advanced Real-Time Methods on the ADE20K Dataset. FPS is measured at a resolution of 512 × 512. Our results are highlighted in red.

Method	Params	GFLOPs	FPS	mIoU
BiSeNet V2	-	118.5	44.9	30.7
RTFormer-S	4.8 M	-	95.2	36.7
RTFormer-B	16.8 M	-	93.4	42.1
SeaFormer-B-Seg100	8.6 M	-	44.5	41.0
PIDNet-M	37.4 M	197.4	28.6	39.6
AFFormer-B-Seg	3.0 M	-	49.6	41.8
DMPNet	0.74 M	259.7	-	35.2
DSNet	6.5 M	226.6	72.3	40.0
LCFNet	7.3 M	77.92	59.3	37.6
Ours	19.8 M	158.7	143.6	42.7

**Table 4:** Comparison with other state-of-the-art real-time methods on the COCO-Stuff-10K test set. FPS is measured at a resolution of 640 × 640. Our results are highlighted in red.

Method	Params	GFLOPs	FPS	mIoU
BiSeNet V2	-	118.5	38.7	25.4
DDRNet-23	20.1 M	143.1	108.8	32.1
SeaFormer-B-Seg100	8.6 M	-	41.9	34.1
AFFormer-B-Seg	3.0 M	-	46.5	35.1
DSNet	6.3 M	77.92	57.9	31.8
LCFNet	5.9 M	226.6	-	33.7
Ours	18.3 M	158.7	133.5	35.4

**Table 5:** Ablation Study of Each Component.

LALK	MSPA	MSCNT	mIoU
✓	✓		79.61
		✓	78.60
✓		✓	79.39
✓	✓	✓	80.05

**Table 6:** Ablation study of MSCNT modules.

LALK	MSPA	MSCE	NTM	FPS	mIoU
√	√	√		78.1	79.68
√	√		√	77.5	79.81

**Table 7:** Performance of NTM module under different noise scenarios.

Noise	LALK	MSPA	MSCE	NTM	mIoU
High	√	√	√	√	79.91
Low	√	√	√	√	79.87

**Table 8:** Ablation study of DWConv.

Type	MSPA	MSCNT	mIoU
DWConv	√	√	79.93
Conv	√	√	77.67

**Table 9:** Ablation on different types of loss.

Loss Type	Baseline	KL Loss	MI Loss	L2 Loss	CWD Loss
mIoU (%)	78.5	78.3	79.6	77.3	79.6

**Table 10:** Ablation on the location of alignment loss.

Logits	Stage 2	Stage 4	Decoder	mIoU (%)
				78.2
√				78.5
√	√			79.2
√	√	√		79.5
√	√	√	√	79.6

**Table 11:** Ablation on the weight of alignment loss.

Loss Weight	0,0,0,0	3,0,0,0	3,5,5,5	3,10,10,10	3,15,15,15
mIoU (%)	78.5	78.8	79.4	79.7	79.3

**Table 12:** Ablation on the convolution kernel size.

Kernel Size	FPS	nIoU (%)
1 × 1 × 1 + 1 × 1 × 1	75.9	78.4
1 × 1 × 1 + 5 × 5 × 5	74.6	78.7
5 × 5 × 5 + 5 × 5 × 5	73.3	79.0
5 × 5 × 5 + 7 × 7 × 7	72.8	79.8
1 × 1 × 1 + 7 × 7 × 7	74.9	79.3
7 × 7 × 7 + 7 × 7 × 7	69.8	78.8

## Data Availability

This study uses publicly available datasets to support its findings. The Cityscapes dataset is available at https://www.cityscapes-dataset.com (accessed on 1 October 2023), the ADE20K dataset can be accessed at https://groups.csail.mit.edu/vision/datasets/ADE20K/ (accessed on 1 October 2023), and the COCO-Stuff 10K dataset is available at https://github.com/nightrome/cocostuff10k (accessed on 1 October 2023). All datasets are freely accessible for academic research under their respective licenses.

## Abbreviations

The following abbreviations are used in this manuscript:

LKNTNet	Large Kernel CNN Network Including a Noise Transfer Mechanism
LALK	Location-Aware Large Kernel
MSPA	Multi-Scale Position-Aware Block
MSCNT	Multi-Scale Contextual Noise Transfer
NTM	Noise Transition Matrix
